# Connecting plant, animal, and human health using untargeted metabolomics

**DOI:** 10.1093/jas/skaf254

**Published:** 2025-08-16

**Authors:** Anita Fleming, Fred D Provenza, Frédéric Leroy, Stephan van Vliet, Catherine Elliot, Michael Hamlin, Konagh Garrett, Cameron Joel Marshall, Pablo Gregorini

**Affiliations:** Department of Agriculture and Life Sciences, Lincoln University, Lincoln, Christchurch, New Zealand; Department of Wildland Resources, Utah State University, Logan, UT; Research Group of Industrial Microbiology and Food Biotechnology (IMDO), Faculty of Sciences and Bioengineering Sciences, Vrije Universiteit Brussel, Brussels, Belgium; Department of Nutrition, Dietetics & Food Sciences, Utah State University, Logan, UT; Department of Agriculture and Life Sciences, Lincoln University, Lincoln, Christchurch, New Zealand; Department of Agriculture and Life Sciences, Lincoln University, Lincoln, Christchurch, New Zealand; Department of Agriculture and Life Sciences, Lincoln University, Lincoln, Christchurch, New Zealand; Department of Agriculture and Life Sciences, Lincoln University, Lincoln, Christchurch, New Zealand; Department of Agriculture and Life Sciences, Lincoln University, Lincoln, Christchurch, New Zealand

**Keywords:** cattle, grazing management, health, human, metabolome

## Abstract

Emerging evidence suggests that the metabolites present in biochemically diverse herbages cascade across trophic levels, influencing both the meat quality of grazing cattle and human metabolomic profiles. This study compared the metabolomic profiles of Angus cattle finished on three distinct pasture systems: a standard perennial ryegrass and white clover sward (PRG), a complex multispecies mixture (CMS; *n* = 22 species), and adjacent monoculture strips (AMS) comprising ryegrass, chicory, plantain, lucerne, and red clover in equal areas. The resulting tenderloins were processed into (250 g) beef patties and assessed in a double-blind, randomized, crossover clinical trial involving 23 human participants (ANZCTR registration: ACTRN12624001081505). The AMS herbage contained higher concentrations of gamma-tocopherol (vitamin E) and eicosapentaenoic acid (EPA), which were reflected in elevated levels of these compounds in the beef (*P *< 0.05) and, subsequently, in human plasma 3 to 5 h *postprandial (P < *0.05). These results are the first to demonstrate that human metabolomic responses are directly influenced by the forage composition of grazing cattle, highlighting a novel linkage between pastoral diversity, animal diet, and consumer health outcomes.

## Teaser

From pasture to plate to person—what cattle eat shapes the beef and the body.

## Introduction

Consumers and farmers are increasingly aware of the intricate connections between the health of the land, animals, and humans, as well as of our collective responsibilities to sustain and enhance Te Taiao (meaning the natural world in Te Reo Māori), for future generations (Gregorini and Maxwell, 2020). Consequently, land users, policy-makers, and wider society are demanding alternative approaches to ensure environmental sustainability, food security, and enhanced animal wellbeing from pastoral agriculture (Schiere and Gregorini, 2023). There is growing demand for agroecological systems that are functionally diverse, adaptive, and integrative, operating across multiple spatial and functional scales—referred to as *multiscapes*. These multiscapes extend beyond traditional landscapes to encompass interconnected sources of food (*foodscapes*), health (*healthscapes*), and cultural values (*socialscapes*), collectively functioning as sustainable and resilient ecosystems (FAO, 2018; Provenza et al., 2021; Gregorini et al., 2022).

Short-term, postprandial inflammation has been linked to cardiometabolic diseases such as obesity, type-2 diabetes, cardiovascular disease, cancer, and metabolic syndrome. Several epidemiological studies have associated increased postprandial inflammation with the consumption of red meat and dairy products (Chan et al., 2011; Micha et al., 2012). As a result, some individuals and organisations aiming to improve health outcomes are turning toward dietary patterns that exclude animal-source foods. While the scientific community is trying to resolve ongoing controversies on whether animal-source foods are causally related to cardiometabolic disease (Barnard and Leroy, 2020). A critical gap in the understanding of nutrition and human health pertains to the large biochemical heterogeneity within the broad categories of meat and dairy coming from different production systems—and the diets used to feed livestock (e.g., grazed pastoral species or ration-based diets). The question arises as to whether there are ecological implications and connections between phytochemically diverse swards and the health of humans and soil (Provenza et al., 2019). If so, this expanded pool of nutrients, medicines, root exudates, and prophylactics, known as plant secondary metabolites or phytochemicals, must be considered to understand how consuming plant phytochemicals that are concentrated in the meat and milk of livestock, can impact human metabolism (van Vliet et al., 2021; Marshall et al., 2022).

The phytochemical profile of forages, grasses, and forbs grazed by ruminant livestock is diverse. Several variations of phenolic compounds (e.g., phenolic acids, flavonoids, and complex tannins), as well as terpenoids (Mono-, di-, tri-, and sesqui-terpenoids), alkaloids, and glucosinolates can exist within a single plant. For example, there are over 90 of these compounds present in chicory (*Chicorium intybus*) (Fleming and Gregorini, 2023), many of which have antioxidant, anti-inflammatory, anti-cancer, and heart-health-promoting effects in humans (Bulló et al., 2007; Thomasset et al., 2009; Khoo et al., 2017; Smeriglio et al., 2017; Dludla et al., 2018; Platosz et al., 2020; Fleming and Gregorini, 2023). Certain plant phytochemicals can also reduce metabolic and physiological stress in grazing ruminants, increase animal performance, feed conversion efficiency (Beck and Gregorini, 2020; van Vliet et al., 2021; Garrett et al., 2021a, b, 2022), and enhance the hedonic and eudemonic wellbeing of livestock (Beck and Gregorini, 2020, 2021; Marshall et al., 2021; Garrett et al., 2021a, b). However, research has not yet established a clear link between plant phytochemicals, livestock, and human metabolism due to the vast diversity of phytochemicals present in pastoral plant species and a limited understanding of their metabolism across different trophic levels.

Metabolomic techniques provide a comprehensive analysis of low-molecular-weight metabolites (amino acids, vitamins, minerals, carbohydrates, sugars, lipids, fatty acids, phenolic compounds, and alkaloids) that depict basic metabolic pathways of a biological system (Gibney et al., 2005; Alonso et al., 2015). In livestock production, metabolomic profiling has been used as a “fingerprinting” technique to give product credence and describe how diet or genotype alters the metabolism of livestock (Saleem et al., 2012; Carrillo et al., 2016; Marshall et al., 2022). However, in this experiment, untargeted metabolomics was employed to map metabolic pathways across multiple trophic levels (e.g., plant, animal, human), enabling a novel connection between the human consumer and both the beef they consumed and the specific forages that contributed to its production.

The objective of this work was to explore, for the first time, the effect of taxonomically and phytochemically rich, functional swards-as opposed to simple mixes of perennial ryegrass (*Lolium perenne*) and white clover (*Trifolium repens*) on plant, animal, meat, and human metabolism, by assessing the metabolomic profiles of plants, animals, meat, and humans (Kronberg et al., 2021). In other words, this research begins to address critical knowledge gaps concerning the phytochemical diversity of herbages used to graze livestock and their subsequent effects on the metabolism of human consumers (Provenza et al., 2019).

## Materials and Methods

All procedures outlined were approved by the Lincoln University Animal Ethics Committee (AEC:2021-10) and Human Ethics Committee (HEC 2021-36). The clinical trial was conducted following the Declaration of Helsinki Principles, although the trial was retrospectively registered with ANZCTR on 6 September 2024, registration number: ACTRN12624001081505.

### Beef finishing trial

This study was conducted at the Lincoln University sheep farm, Canterbury, New Zealand (−43.644601°N, 172.451242°E), to evaluate liveweight gain, oxidative stress, estimated dry matter intake (DMI) of growing cattle over 120 d between October and February of 2021–2022, during summer months. The experiment was run as a complete block design involving three grazing treatments.

During the trial, 30, 2-yr-old, mixed-sex (heifers = 15, steers = 15), Angus cattle (315 ± 35 kg liveweight) were grouped into triplicates based on their sex, liveweight, and date of birth, and randomly allocated to one of three forage treatments (*n* = 10 per forage treatment). The three dietary treatments consisted of a perennial ryegrass-based sward (PRG), a complex multispecies mixture (CMS, *n* = 24 forage species) including grasses (PRG, Italian ryegrass: *Lolium multiflorum*, tall fescue: *Lolium arundinaceum*, meadow fescue: *Schedonorous pratensis*, prairie grass, *Bromus wildenowii*, timothy *Phleum partense*, and cocksfoot: *Dactylis glomerata*), legumes (white clover: *T. repens*, red clover: *Trifolium pratense*, alfalfa: *Medicago sativa*, lotus: *lotus pedunculatus*, lupins: *Lupinus* and vetch: *Vicia sativa* as well as crimson: *Trifolium incartum*, balansa: *Trifolium michelianum*, persian: *Trifolium resupinatum* and strawberry clovers: *Trifolium fragiferum*), in addition to brassicas (rape: *Brassica napus* and radish: *Raphanus sativus*) and herbs (chicory: *Cichorium intybus* and plantain: *Plantago lanceolata*). The third treatment consisted of a functionally diverse sward, as described previously (Garrett et al., 2022), containing five adjacent monoculture strips (AMS) of perennial ryegrass, red clover, plantain, chicory, and alfalfa that were sown longitudinally across the paddock in equal-sized areas. Cattle were born in the spring of 2020 (October to November), and following weaning, grazed a traditional pastoral diet consisting of perennial ryegrass and white clover at the Lincoln University Ashley Dene Station, Canterbury, New Zealand −43.645105°N, 172.332991°E.

The total grazing area available consisted of 9.5 ha split across 10 paddocks ranging from 0.7 to 1.5 ha in size. The paddocks were randomly assigned a treatment forage mixture, which was established by direct drilling on the 23rd of April 2021. Each treatment mob of cattle grazed the paddocks (*n* = 4 per treatment) rotationally between the 10th of October and the 16th of February 2021 before slaughter at a commercial abattoir in accordance with the Ministry of Primary Industries of New Zealand. Each mob was allocated the same amount of herbage dry matter (DM) calculated to exceed metabolic requirements for each animal based on their average liveweight (i.e., ~4% liveweight per day). The break size of each treatment was calculated by the available herbage mass (pre-grazing herbage mass − post-grazing herbage mass) with a target post-grazing herbage mass of 1,700 kg DM/ha. Cattle were confined to the calculated break area using a back fence and always had free access to water.

Herbage mass was estimated pre- and post-grazing by measuring all plant material collected within a 0.2 m^2^ quadrant as previously described (Fleming et al., 2018). Quadrants were randomly placed at two locations of each strip of the AMS paddocks, and 6-10 locations within paddocks of PRG and CMS. The average difference between pre-and post-grazing herbage mass (i.e., herbage disappearance) was used to estimate daily DMI from the area available for grazing. Botanical and chemical composition and DM percentage of each diet were evaluated monthly, and sample collection, sub-sampling, and processing methods were adapted from Fleming et al. (2018, 2020), with the exception that a third subsample of each herbage was collected for metabolomic analyses. Herbage samples were stored at −20 °C (for up to 4 mo) until freeze-dried and ground through a 1-mm screen (ZM200 Retsch) to analyze metabolomic profiles and chemical components (water-soluble carbohydrate [WSC], organic matter [OM], DM, dry organic matter digestibility, and crude protein [CP]) by near-infrared spectrophotometry (NIRS; Model: FOSS NIR Systems 5000, Maryland, USA; calibration *r* > 90 %). A subsample (5 g) of dried and ground herbage of each sward was pooled from each measurement to evaluate the metabolomic profile. Freeze-dried plant material was stored in a cool dark location until analyzed using untargeted metabolomics ~6 mo following sample collection.

### Animal measurements

Each month, on the third day following the allocation of a new pasture break, cattle were removed from their experimental pastures and herded to a crush where they were weighed (Tru-test, Texas, USA). A sample of blood was collected from the coccygeal vein in two 10-mL vacutainers (with either lithium heparin or EDTA as the coagulant) and immediately stored on ice. Following collection, blood was transported to a laboratory, and the plasma was separated by centrifuging the samples at 3000 × *g* for 12 min. The plasma was pipetted into 1.5-mL Eppendorf containers and stored at −20 °C until analyzed. Plasma collected from Li heparin-coated vacutainers was analyzed for total antioxidant status (TAS: Cat No. NX2332), while that collected from EDTA-coated vacutainers was analyzed for non-esterified fatty acids (NEFA: Cat No. FA115) using a Randox RX Daytona clinical analyzer (Randox Laboratories Ltd, Crumlin, UK) following their respective kit instructions. Blood samples and liveweights were collected on day 1 of the experiment and then each month after to allow for 30-d acclimatization to experimental diets.

The cattle grazed the experimental diets until 16 February 2022, when they were transported to a local commercial abattoir, weighed, and then slaughtered. Following slaughter, meat quality/carcass (carcass yield, carcass weight, temperature, pH, marbling, meat color, fat color, rib fat, and ossification) characteristics of each animal were evaluated using the Silver Fern Farms Eating Quality System (https://silverfernfarms.com/nz/en/what-were-made-of/guaranteed-eating-quality). One cut from the *psoas major* muscle of each animal was ground (5-mm mincing plate), sub-sampled, homogenized by treatment to create a consistent product, and processed in a commercial kitchen into beef patties weighing 250 ± 5 g consisting of 100% beef (Food South, South Island Hub, Canterbury, New Zealand). Patties were blast-frozen and stored at −20 °C. The subsample of beef collected from each animal was stored at −20 °C until analyzed using metabolomic methods.

### Human postprandial trial

Between May and July 2022, a three-way, double-blind, randomized crossover trial was conducted at Lincoln University (New Zealand) Exercise Science Laboratory (-43.644828, 172.465163, Canterbury, New Zealand).

Twenty-three individuals in total (*n* = 16 female, age = 58 ± 13 yr, and 7 = male, age = 53 ± 11.9 yr old) with cardiometabolic disorders were selected to participate in a randomized crossover experiment. The trial was conducted using two cohorts of participants (*n* = 12 each) over 6 wk (2- × 3-wk periods). During each 3 wk, participants were randomly assigned a sequence of each beef treatment (double-blind). Each week, participants were offered two ~250 g (486 g raw weight) beef patties produced from the cattle that grazed the three treatments. During each visit, baseline, and postprandial measurements (at 0-, 3-, and 5-h post-meal) of plasma glucose, cholesterol profiles, C-reactive protein (CRP), blood pressure, and metabolomic profiles were collected to evaluate the response to a single meal of beef. A wash-out period of 7 d was used between visits (i.e., feeding) until each participant had consumed all three beef treatments. The evening before each visit, participants consumed a meal of pasta (300 g) and vegan pesto, which was prepared and delivered by Pasta Vera (Wigram, Christchurch, New Zealand). This was selected to prevent residual dietary effects on metabolomic and metabolic samples the following day and provide a simple standardized diet. Four participants were assessed in a day over 3 d each week, and they were asked to abstain from smoking, alcohol, and eating after dinner the night before the consumption of beef. Each participant assessed and offered the beef meal individually, with a 30-min gap between the arrival of each person for the collection of baseline measurements. Participants arrived at the laboratory for baseline measurements between 07:30 and 09:30 h each day.

Patties were thawed at 4 °C for 36 h before cooking. During each visit, 486 g of raw ground beef was cooked (mean = 341 g cooked meat per person) using an electric fry pan (temperature controlled) with no added oil or salt until each patty reached an internal temperature of 71 °C (approximately 5.5 min each side). The fry pan was thoroughly cleaned between each patty.

During the first measurement, each participant completed a physical activity survey (IPAQ-SF) and a body composition scan via an 8-electrode bioelectrical impedance (Accuniq B380), followed by blood pressure and carotid–femoral pulse wave velocity (tonometry) (Sphygmocor EXCEL) tests on arrival. Resting venous blood samples were collected by a registered nurse from the antecubital vein using standard guidelines. Resting blood samples and blood pressure measurements were taken on arrival prior to meat consumption, and following baseline measurements, each participant was given up to 15 min to consume the meat allocated, or until they were satiated. Plasma samples for metabolite analyses were collected in an EDTA-coated vacuette and centrifuged at 5000 RPM for 10 min at 4 °C. Plasma was pipetted into 2-mL plastic microtubules and stored at −20 °C until analyzed. Participants were asked to remain sedentary (i.e., seated with no movement other than viewing an electronic device, or reading) without food (allowed to drink water) for 5 h with blood pressure and blood sample collections repeated at 3- and 5-h postprandial. After the final measurement, participants were offered a wide variety of snacks to eat prior to departure.

### Nontargeted metabolomics

#### Sample extraction and preparation

##### Lipidomics.

Samples of plant, meat, and human plasma were extracted using butanol: methanol (1:1) based on the method of Huynh et al. (2019). Solvents were Optima LCMS grade from ThermoFisher (Auckland, New Zealand).

##### GCMS/MS and semi-polar metabolites.

Freeze-dried, ground (1 mm, as previously described), then frozen plant and meat samples were further ground using a bead mill (TissueLyser II, Qiagen) and a metal bead, and were shaken for 5 min at 30 Hz. A powdered sample of plant or meat (10 or 25 mg, respectively) was weighed into a 2-mL microcentrifuge tube, with 10 µL of internal standard mix (Splash Lipidomix, Avanti Polar Lipids, Alabaster, Alabama, USA) and 990 µL of butanol: methanol (1:1 v/v) with 5 mM ammonium formate. Ten microliters of human plasma was pipetted into a 2-mL microcentrifuge tube, and 5 µL of internal standard mix and 95 µL of butanol: methanol with 5 mM ammonium formate. A metal bead was added to the microcentrifuge tube, and plant, meat, and plasma samples were shaken in a bead shaker for 5 min at 30 Hz. The samples were then sonicated for 60 min at 20 °C before centrifugation (14,000 × *g* for 10 min at 20 °C, Heraeus Megafuge 8R, ThermoFisher). Plant (150 µL), meat (200 µL), or human plasma (80 µL) supernatant was aliquoted into an amber chromatography vial with a 250-µL flat-bottom insert, and 150 µL supernatant was taken from each sample to make a pooled quality control sample used to check for instrument variation.

The extraction procedure for semi-polar metabolites was based on that of Jiye et al. (2005). Fifty or 25 mg of powdered pasture or meat (respectively) or 100 µL of plasma was transferred to a 2-mL microcentrifuge tube and 980 µL of methanol: water (9:1 v/v), 20 µL of an in-house stable isotope internal standard mix (citric acid d4, fumaric acid d4, succinic acid d6, U13C-glucose, L-dopa d3, benzoic acid d5, stearic acid d35, lauric acid d23, cholic acid d5, all from Cambridge Isotopes Ltd, Tewksbury, MA, USA, at a concentration of 128 µg/mL), and a metal bead were added. Plant, meat, and plasma samples were extracted in a bead shaker (5 min, 30 Hz), and tubes were transferred to a −20 °C freezer for 30 min. The extracts were centrifuged for 10 min at 14,000 × *g* at 4 °C. A 180 µL sample of the supernatant was transferred to an amber chromatography vial with a tapered insert for GC-MS/MS analysis, 180 µL transferred to an amber chromatography vial with a flat-bottomed insert for semi-polar metabolomics analysis, and 180 µL transferred to a 15 mL Falcon tube for a pooled QC sample.

Samples for GC-MS/MS were dried in a vacuum centrifuge concentrator (Christ RVC 2-18 CD plus, Martin Christ) and derivatised by methoxymation (30 µL of 30 mg/mL methoxyamine hydrochloride, (Sigma-Aldrich, Auckland, New Zealand) in pyridine, incubated for 16 h at room temperature) and silylation (30 µL of N-methyl-N-(trimethylsilyl)trifluoroacetamide + 1% trimethylchlorosilane) (ThermoFisher, Auckland, New Zealand) incubated at 50 °C for 60 min, before addition of 15 µL of 30 µg/mL methyl nonadecanoate (Sigma-Aldrich) in heptane (Savolainen et al., 2016).

#### Metabolomic analysis

Sample extracts were analyzed using untargeted lipidomics, untargeted semi-polar metabolomics, and targeted GC-MS/MS metabolomics. All samples were run using similar batch sequences, consisting of 4 blank injections, 5 sample preconditioning injections, 1 quality control sample, 10 samples, 1 quality control sample, 10 samples, and so on. Each batch included an extraction blank including internal standards (microcentrifuge tube with no sample and extracted as for the samples), and an extraction blank with no internal standard (as before, but no internal standard added before extraction). Samples were run in randomized order using the random number generator in Excel. As the human plasma samples were repeated measures from the same subject, samples were first randomized based on subject, and then repeated samples were randomized within each subject.

### Lipidomics analysis

Lipidomics analyses were carried out using LC-qToF-MS (LCMS 9030, Shimadzu, Kyoto, Japan) in data-independent analysis (DIA) mode. Samples (2 µL) were injected into a C18 column (Acquity CSH-C18, 100 × 2.1 mm, 1.7 µm, Waters, Milford, MA, USA). Lipid classes were separated using gradient chromatography. Mobile phaseSolvent A was water/isopropanol/acetonitrile (50:30:20 v/v/v) with 20 mM ammonium acetate, while solvent B was water/isopropanol/acetonitrile (1:90:9 v/v/v) with 20 mM ammonium acetate. All solvents and additives were LC-MS grade. The gradient program was as follows: starting, 10% B, 2.7 min, 45% B; 2.8 min, 53% B; 9 min, 65% B; 9.1 min, 89% B; 11 min, 92% B; 11.1 min, 100% B; 13.9 min, 100% B; 14 min, 10% B; 17 min, 10% B. The flow rate was 0.4 mL/min. The column oven was set to 60 °C and autosampler set to 20 °C and data were acquired using positive electrospray ionisation The DIA method was as follows: ToF scanning between m/z 250 and 1,300 with an event time of 0.03 s, and 41 windows of m/z 20 from m/z 300 to 1,100, in MS/MS mode with a collision energy of 23 eV and a collision energy spread of 6 eV, and event time of 0.02 s. LC-MS interface settings were as follows: nebulising gas flow 2 L/min; heating gas flow 10 L/min; interface temperature 300 °C, interface voltage 4.5 kV in positive mode and −3.5 in negative mode, desolvation line temperature 250 °C, heat block temperature 400 °C.

### Semi-polar metabolomics

Semi-polar metabolomics analyses were carried out using LC-qToF-MS (LCMS 9030, Shimadzu, Kyoto, Japan) in DIA mode as above. Samples (4 µL) were injected into a C18 column (Hypersil Gold C18, 100 × 2.1 mm, 1.9 µm particle size, ThermoFisher). The chromatography was set up to run positive and negative ionisation modes alternately, on different columns. Pumps A and B were used for the analytical gradient, and pumps C and D were used for column regeneration. The following analytical gradient was used: Starting, 0% B; 1 min, 0% B; 11 min, 100% B; 16.9 min, 100% B; 17 min, 0% B, 18 min, 0% B. Solvent A was 16 mM ammonium formate in water, solvent B was 0.1% formic acid in acetonitrile, and the flow rate was 0.4 mL/min. The column regeneration program was as follows: starting, 100% B; 12 min, 100% B, 15 min; 0% B, 17 min, 0% B. The column oven was set to 40 °C and the autosampler set to 4 °C. The mass spectrometer was run in DIA mode with an electrospray ionisation source. This was set up as follows: scanning data between m/z 55 and 1,100 in ToF mode with an event time of 0.03 seconds, and 41 windows of m/z 20 from m/z 100 to 900, in MS/MS mode with a collision energy of 23 eV and a collision energy spread of 15 eV, and event time of 0.02 s. Analyses were carried out in both positive and negative ionisation modes, with interface settings as for lipidomics.

#### Gas chromatography-mass spectrometry.

GC-MS analyses were carried out on a GCMS TQ8040 instrument (Shimadzu) using the SmartMetabolites MRM method. This targeted metabolomics method includes 450 multiple reactions monitoring transitions, covering approximately 400 individual metabolites (several metabolites have two chromatographic peaks), mainly relating to central metabolic pathways (e.g., energy metabolism, protein synthesis and breakdown, lipid metabolism). Analysis conditions were injector temperature 250 °C, interface temperature 280 °C, and MS source temperature 200 °C. The chromatography was carried out on a BPX5 column (30 m × 0.25 mm, 0.25 µm film thickness, Trajan, Ringwood, VIC, Australia) with the following temperature gradient: start 60 °C, 2 mins 60 °C, 20 min 330 °C, 23 min 330 °C. A 2 µL derivatized sample was injected in split injection mode with a ratio of 1:30, and carrier gas linear velocity was held constant at 40 cm/s. Peaks were identified based on the ratio of quantifier and qualifier ions and retention index.

#### Data processing and metabolite identification

LC-MS data were processed using MS DIAL v4.80 (Tsugawa et al., 2015), including peak deconvolution, database-based identification, and retention time and mass alignment. In brief, mass accuracy thresholds were 0.01 Da for MS1 for lipidomics and 0.0075 for semi-polar metabolomics, and 0.025 Da for MS2 for both. The minimum peak height for peak detection was 1,000 units, MS2 deconvolution used a sigma window value of 0.5, and an MS/MS abundance cutoff of 0 amplitude. Lipidomics database searching used the built-in lipidomics database within MS DIAL based on MS1 and MS2 matching, and adducts included were [M + H]^+^, [M + NH_4_]^+^, [M + Na]^+^, [M − H]^−^, [M − H_2_O − H]^−^, and [M + Cl]^−^.

Lipidomics identifications were refined based on modelling likely retention times based on a mixture of 69 lipid standards (UltraSplash, Avanti Polar Lipids). Lipid identifications that fell outside a ±10% retention time range were considered to be incorrectly identified. LC-MS lipidomics and semi-polar metabolomics data were manually searched against an in-house library that incorporates the IROA Mass Metabolite Library of Standards (IROA Technologies, Ann Arbor, MI, USA), and additional metabolite standards available in-house. Identification against these standards was based on accurate mass, retention time, and isotopic score using LabSolutions Insight Explore (Shimadzu).

For semi-polar metabolites, the data were searched against combined ESI-MS/MS databases based on authentic standards available via MS DIAL (https://systemsomicslab.github.io/compms/msdial/main.html#MSP). Identification of lipids was further refined based on retention time (see below), while semi-polar metabolite identification based on database matching was treated as tentative. Adducts included for searching were [M + H]^+^, [M + NH_4_]^+^, [M + Na]^+^, [M + CH_3_OH + H]^+^, [M + ACN + H]^+^, [M + H − H_2_O]^+^, [M − H]^−^, [M − H_2_O − H]^−^, and [M + Cl]^−^. Data were aligned to a quality control sample run in the middle of each batch with a retention time tolerance of 0.05 min and MS1 tolerance of 0.015 Da. Blank features were removed based on the extraction of blank samples. Data were normalized using LOWESS normalization based on a quality control sample between every 10 sample injections.

GC-MS data were processed using LabSolutions Insight for GC-MS (Shimadzu), with compounds identified based on retention index and quantifier to qualifier ion ratio.

### Statistical analyses

Cattle performance, apparent DMI, and blood parameters (TAS, PUN, and NEFA) were tested for normality and analyzed by either linear mixed models or generalized linear mixed models using the lme4 package in R studio in R (v.3.4.4., https://www.r-project.org/). Models were fitted by evaluating treatment, sampling date (LW, LWG, DMI, TAS, PUN, and NEFA), and sex and their interactions as fixed effects and using the individual animal as the experimental unit. The baseline blood metabolite profile of individual animals was used as a covariate.

Measurements of cardiovascular function and circulating concentrations of blood metabolites (glucose, CRP, cholesterol, high-density lipoprotein [HDL], and low-density lipoprotein [LDL]) were tested for normality, and treatment effects were evaluated using either linear mixed models or generalized linear mixed models using the lme4 package in R studio. Regression models were developed using treatment, time of sampling, sex, order of treatment eaten, and their interactions as fixed effects, while individuals nested within the day of sampling were considered as the random effect. The significance of univariate statistics was determined if *P* < 0.05, and tendencies were denoted as *P* < 0.1 and >0.05.

Statistical analyses of peak intensities were performed in Metaboanalyst (v.5.0, https://www.metaboanalyst.ca/). Peak intensities were log-transformed (base 10) and normalized by the median of each dataset. Plant and beef metabolomic and lipidomics were assessed using a one-way ANOVA. Plasma metabolomics was analyzed using a combination of two-way ANOVA to evaluate time-series data (treatment × time interactions), and linear models to account for random error of individual people and to enable covariate analysis (sex, age, body mass index, amount of beef consumed). Due to the explorative nature of this study, differences in raw *P*-values were considered significant if *P* < 0.1, while the FDR was considered acceptable if FDR < 0.1 as described previously (Benjamini and Hochberg, 1995; Storey and Tibshirani, 2003). The FDR indicates the probability or proportion of a false discovery or a type I error (the null hypothesis is rejected when in fact it is true) of multivariate testing. Due to the explorative nature of this study, the FDR < 0.1 is regarded as “high confidence” or “statistically significant”, while FDR > 0.1, <0.25 are regarded as “possible” or “hypothesis,” as described previously by others (Zhang et al., 2010; Kim et al., 2015).

## Results

### Herbage composition

The botanical components of each sward are presented in Table 1. The PRG sward contained greater components of grasses (Italian and perennial ryegrass), which were at a greater reproductive state of growth compared with both CMS and AMS diets (40%, 25%, and 11% DM, respectively; *P < *0.01). The percentage of dead material also increased with the PRG diet compared with CMS and AMS (14%, 4%, and 2% DM, respectively; *P* < 0.01). The proportion of weed species such as mellow (*Modiola caroliniana*), shepherd’s purse (*Capsella bursa-pastoris*), barley grass (*Hordeum murinum*), and stinging nettle (*Urtica dioica*) increased in the AMS swards compared with PRG or CMS swards (Table 1). The AMS sward contained a greater percentage of forbs (chicory and plantain) than CMS and PRG (27%, 5%, and 0%, *P* = 0.07, respectively). The clover content of the PRG diet was 16.3% DM, compared with 24.2% and 8.7% observed for CMS and AMS, respectively. The clover content of the swards did not differ statistically, although there was a high variation of clover between paddocks, explaining the high standard error reported for this variable (±6.9%).

**Table 1.** Estimated dry matter intake (DMI), sward conditions and botanical components of perennial ryegrass and white clover (PRG), complex multispecies mixture (CMS), and adjacent monoculture strips (AMS).

Variable	PRG	CMS	AMS	SE	*P*-value	
					Diet	Date
Herbage mass (kg DM/ha)	5078^a^	6144^b^	4671^a^	285.5	0.002	***
Post-grazing mass	2436^a^	2495^a^	2350^a^	129	0.72	***
Apparent allocation (kg DM/cow/d)	25.6^a^	20.2^a^	23.0^a^	2.43	0.33	0.02
DMI	17.2^a^	13.1^a^	16.2^a^	1.33	0.12	0.49
Botanical component						
Grasses vegetative (% DM)	17.3^ab^	12.0^ab^	3.29^a^	4.94	0.07	0.79
Grasses reproductive	40.1^b^	25.0^a^	11.4^a^	14.0	**	0.21
Clovers	16.3^a^	24.2^a^	8.66^a^	6.87	0.17	0.84
Other legumes	0.0^a^	17.4^a^	8.4^a^	5.95	0.21	0.89
Herbs	0^a^	4.52^a^	26.9^ab^	15.9^1^	0.07	0.99
Brassica	0^a^	6.1^b^	0^a^	0.80	***	0.5
Weeds	12.3^ab^	6.7^a^	39.1^b^	7.16	***	0.99
Dead	14.0^b^	4.0^a^	2.4^a^	2.59	**	0.03

Superscripts within rows are significantly different (*P* < 0.05).

*P < 0.05, **P < 0.01, ***P < 0.001.

Pre- and post-grazing herbage masses differed across swards, and interactions between date and type of sward were also identified (*P* < 0.05), reflecting seasonal variation in the growth of the individual components of each treatment. Apparent daily DM allocations and individual animal DMI were similar across treatments (Table 1), although the apparent DMI of the CMS diet was numerically lower than PRG and AMS (13.1 vs. 17.2 and 16.2 kg DM/cow, respectively). The chemical composition of each dietary treatment and the individual monocultures within the AMS are presented in Table 2. All chemical components differed (*P* < 0.05) between individual plants but were not always different across the average overall treatment. The DM content declined with chicory but remained similar across all other plant species and was not different between diets. The NDF content of perennial ryegrass (47% DM) was greater within the individual monocultures of AMS but was reduced by chicory and red clover (30% and 32% DM, respectively), reducing the average NDF content of the AMS sward compared with PRG and CMS (38%, 43%, and 46% DM, respectively). The ADF content followed a similar trend to NDF, except that the ADF content of plantain was greater than ryegrass in the AMS diet (Table 2). The CP content was similar between the PRG and AMS swards but declined 15% with the CMS (Table 2, *P* < 0.001). While the CP content of chicory and plantain was low (<12% DM), the CP of alfalfa and red clover was between 17% and 18% DM. The WSC content of the PRG and CMS swards was similar (18.3% WSC) but declined to 16% (*P* < 0.01) in AMS, reflecting the lower WSC content of red clover and alfalfa compared with chicory and ryegrass (Table 2). The WSC content of the ryegrass offered in the AMS was less than the PRG (15% vs. 18.3%). The DMD of PRG and AMS were similar and declined in the CMS herbage (Table 2).

**Table 2.** Dry matter (DM), neutral detergent fibre (NDF), acid detergent fibre (ADF), organic matter (OM), crude protein (CP), dry matter digestibility (DMD), water-soluble carbohydrates (WSC), and metabolisable energy (ME) percentage of three dietary swards consisting of either perennial ryegrass (PRG), a complex multispecies mixture (CMS), or adjacent monocultural strips of ryegrass, chicory, plantain, alfalfa, and red clover (AMS).

Plant	DM %	NDF %	ADF %	OM %	CP %	DMD	WSC %	ME (MJ/kg DM)
PRG	23.5^a^	43.2^b^	27.7^ab^	91.8^b^	14.7^ab^	70.8^b^	18.3^b^	10.8^b^
CMS	21.6^a^	46.1 ^b^	30.1^b^	92.2^b^	12.5^a^	66.9^ab^	18.3^b^	10.3^a^
AMS average	19.8^a^	37.9^a^	28.7^ab^	91.4^ab^	14.6^ab^	68.7^b^	15.4^ab^	10.5^a^
AMS—Ryegrass	25.3^b^	47.7^b^	29.6^b^	92.2^b^	14.7^ab^	67.6^b^	15.0^ab^	10.4^a^
AMS—Chicory	13.9^a^	30.8 ^a^	26.6^a^	90.1^a^	13.2^ab^	73.7^b^	20.7^b^	11.0^b^
AMS—Plantain	19.9^b^	35.5 ^a^	31.5^b^	92.0^b^	10.7^a^	63.9^a^	14.9^a^	9.8^a^
AMS—Lucerne	20.8^b^	37.8 ^a^	27.3^a^	92.0^b^	7.0^b^	70.5^b^	13.1^a^	10.8^b^
AMS—Red clover	18.9^b^	32.1 ^a^	25.4^a^	91.5^ab^	18.9^b^	72.6^b^	14.7^a^	11.1^b^
SE	1.05	2.27	0.92	0.32	0.94	1.39	1.15	0.182
*P*-Value Plant	***	***	**	**	***	***	**	***

The average composition of the AMS diet was determined by evaluating equal DM proportions of each herbage. Chemical components of individual plants offered in the AMS diet are also presented in this table. Standard error of the mean (SE) is also presented along with *P*-values of diet and date.

^a, b^Superscripts that differ within columns are significantly different, *P *< 0.05.

* *P* < 0.05, ** *P < *0.01, *** *P* < 0.001.

### Animal performance and carcass characteristics

The average liveweight of the cattle grazing CMS was 4% less compared with PRG and AMS (Table 3). The interaction between day and treatment was significant (*P* < 0.001), reflecting an increase in average animal liveweight in January. Heifers were (*P* = 0.06) heavier than steers by 10.7 kg across all treatments. The average daily liveweight gain (LWG) of animals grazed on AMS was 8% and 15% greater compared with those grazing PRG and CMS, although significant Diet × Day interactions were also observed (Table 3). Both TAS and NEFA declined (*P < *0.1) in animals grazing CMS compared with PRG or AMS (Table 3). The plasma concentration of NEFA was 7% greater in heifers compared to steers (*P* < 0.05).

**Table 3.** Average liveweight (LW), daily liveweight gain (LWG), total antioxidant status (TAS), and non-esterified fatty acids (NEFA) of cattle fed either perennial ryegrass (PRG), a complex multispecies mixture (CMS), or adjacent monoculture strips of five plant species (AMS).

Variable		Treatment				*P*-value			
	Sex	PRG	CMS	AMS	SE	Treat	Day	Sex	T × D
LW (kg)	H	382 ^a^	372 ^b^	386 ^a^	3.17	0.03	***	0.06	***
	S	373 ^a^	363^a^	377 ^a^	3.28				
LWG (kg/day)	H	1.21^a^	1.11^b^	1.31 ^a^	0.042	0.01	***	0.18	***
	S	1.13 ^a^	1.02 ^b^	1.23 ^a^	0.043				
TAS	H	1.03 ^ab^	1.01 ^a^	1.03 ^ab^	0.011	0.08	***	0.31	0.06
	S	1.04 ^ab^	1.02 ^a^	1.03 ^ab^	0.013				
NEFA	H	0.22 ^b^	0.16 ^a^	0.20 ^b^	0.019	0.06	**	*	0.16
	S	0.32^c^	0.22^b^	0.28^c^	0.028				

Significant P-values of Treatment, Day, Sex, and T × D are indicated.

*P < 0.05, **P < 0.01, ***P < 0.001.

^a–c^Superscripts that differ within rows (variables) are significantly different, *P* < 0.05.

Carcass characteristics are presented in Table 4. The slaughter liveweight of animals grazing on AMS was 10% less than those fed PRG, and greater than CMS (Table 4). Compared with PRG and CMS, the hot carcass weight of AMS cattle was 11% and 6% lighter, respectively (*P* = 0.03). Meat color (redness of the meat) increased with both the CMS and AMS treatments compared with PRG (*P *= 0.02). Ossification increased (*P *= 0.08) by 9% and 17%, respectively, in CMS and AMS animals compared with those fed PRG.

**Table 4.** Carcass characteristics and final liveweight of cattle finished on either a perennial ryegrass pasture (PRG), a complex multispecies mixture (CMS), or adjacent monoculture strips of five plant species (AMS).

Variable	PRG	CMS	AMS	SE	Treatment	Sex	T × S
Final Liveweight kg	440^b^	408^ab^	394^a^	12.2	0.02	0.42	0.33
**Carcass yield** %	53.7	54.3	53.2	0.48	0.25	0.32	0.62
Hot carcass weight kg	236^b^	222^ab^	209^a^	7.02	0.03	0.64	0.27
pH	5.64	5.86	5.72	0.105	0.29	0.79	0.42
Temperature **°C**	7.63	7.47	7.54	0.206	0.75	0.30	0.20
Marbling	303	308	312	10.4	0.85	0.59	0.95
Meat color	3.21^a^	4.32^b^	4.10 ^a^	0.263	0.02	0.15	0.42
Fat color	1.83	1.20	1.40	0.258	0.23	0.90	0.60
Rib fat **mm**	4.04	3.58	4.20	0.658	0.80	0.47	0.81
Ossification	118^a^	130^ab^	142^ab^	7.35	0.08	0.46	0.92

Significant *P*-values of Treatment, Day, Sex, and T × S are reported.

^a–c^Superscripts that differ within rows (variables) are significantly different (*P* < 0.05).

#### Human feeding trial

The amount of meat consumed was not affected by treatment, although men consumed more than women (296 ± 21.7 vs. 197 ± 6.6 g). Diet-by-time interactions were not significant (*P* > 0.1, Supplementary Table 1). Plasma concentrations of glucose, HDL, CRP, diastolic blood pressure (DBP), triglyceride, and cholesterol were not affected by treatment (*P* > 0.1, Supplementary Table 2). Systolic blood pressure increased (*P *= 0.04) in people who ate AMS beef compared with those who ate PRG beef. The DBP of those who ate CMS beef was intermediary (Supplementary Table 2). Circulating concentration of LDL tended (*P *= 0.06) to increase in people fed AMS beef compared with those fed PRG or CMS beef. The ratio of total cholesterol to HDL increased following the consumption of AMS beef compared with CMS beef, while PRG-raised beef was intermediary (Supplementary Table 2).

#### Plant metabolomics

**Figure 1.**
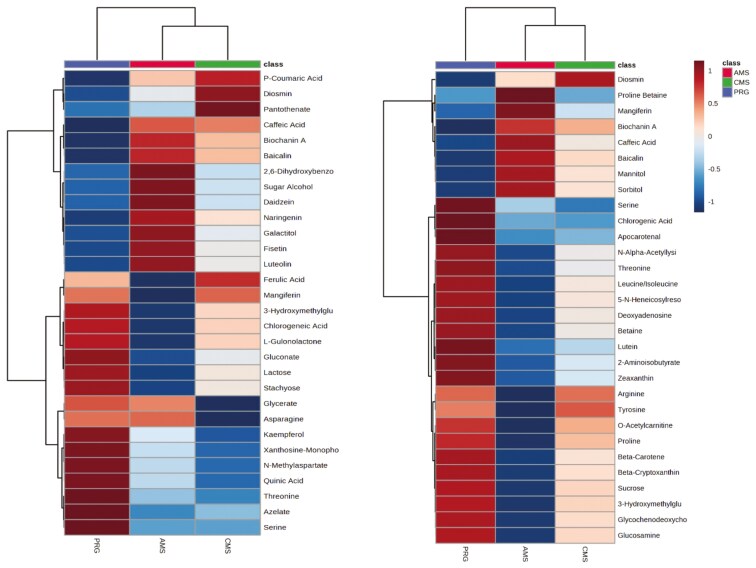
Left: Top 30 compounds (not listed in order) identified by semi-polar metabolomics run in negative mode in plant material. Right: Top 30 compounds identified by semi-polar metabolomics run in positive mode. Plant material was collected from three pastures consisting of either a perennial ryegrass-based (PRG), complex multispecies mixture (CMS), or adjacent monoculture strips (AMS). Differences in fold change are indicated by the colored legend.

A total of 70 features were identified when semi-polar metabolomics was run in positive ionisation mode, 23 differed (raw *P-*value < 0.1) by treatment, and three met multivariate testing criteria (FDR < 0.05). The top 30 (with the greatest fold change across treatments) are presented in Figure 1. Of metabolites to note, AMS increased the relative intensity of proline betaine, biochanin A, an isoflavonoid, and baicalin, a flavone glycoside (FDR < 0.1).

A total of 166 metabolites were identified from GC-MS/MS analysis, of which 49 differed by treatment based on raw *P*-values <0.1, and 27 of these were below FDR < 0.1. These compounds were dicarboxylic acids, sugar acids, phenylacetic acids, TCA acids, and amino acids. Other metabolites of interest were gamma-tocopherol, which increased in the AMS diet (*P *< 0.0001, FDR < 0.0001). Of the lipids identified from lipidomics run in negative mode, 1,651 features were detected, and of those, 45 were positively matched to the metabolite library. Many were neutral glycosphingolipids, ceramides, and glycerophospholipids. All, except one ceramide, increased in AMS and CMS meat compared with PRG. One hundred and eighty-six features were identified by lipidomics in negative mode. Of these, 65 differed with treatment (*P* < 0.1), 5 of these metabolites achieved FDR < 0.1, while the remaining metabolites were “hypothetical” (FDR = 0.28). Of the treatment differences identified with “high confidence,” all were increased in the AMS and CMS swards compared with PRG and were subclassed as ceramides, neutral glycosphingolipids, phosphatidylinositol, and cardiolipin. Some of these were sterols, triacyl- and diacyl-glycerides, and glycerophosphocholine.

#### Beef metabolomics

**Figure 2.**
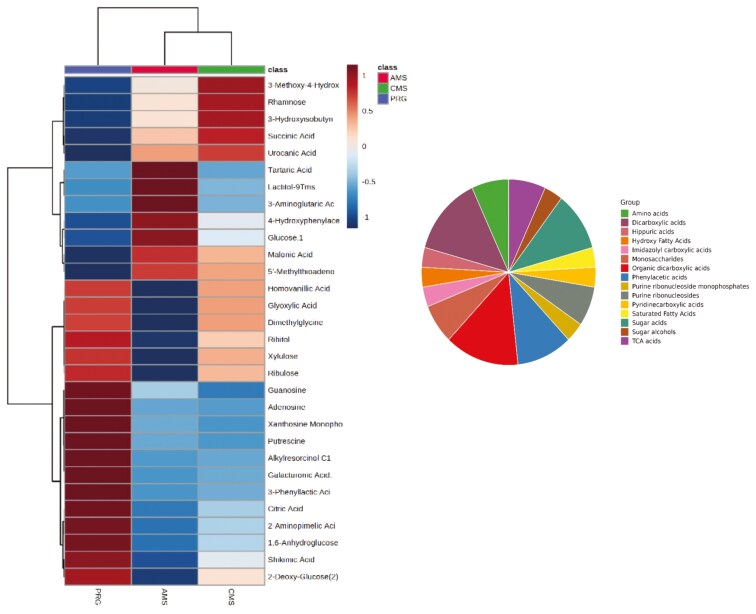
The top 30 metabolite features identified from GCMS/MS of beef produced from cattle finished on either Perennial ryegrass pasture (PRG), a complex multispecies mixture (CMS), or adjacent monoculture strips (AMS). Differences in fold change are indicated by the color bar a fold difference >1.5 is generally considered significant, but refer to text and tables for specifics. The graph indicates subclasses of the metabolites that differed between treatments.

A total of 154 metabolites were detected in meat by GC-MS/MS targeted metabolomics. Twenty-eight metabolites were different (raw *P* < 0.1) between AMS, CMS, and PRG (Figure 2). Following FDR correction, only linoleic acid differed (FDR < 0.007), increasing in meat produced from AMS compared with CMS or PRG (Table 5). Several other metabolites increased in AMS beef, in which raw *P*-values were significant (*P* < 0.01), and FDR values suggest them as “possible” (FDR = 0.14). These were hypoxanthine, pantothenic acid (PA), ribulose-5-phosphate (R5P), and eicosapentaenoic acid (EPA), which were all elevated in AMS beef compared with CMS or PRG beef (Table 5). Of the other metabolites that differed, but did not meet the FDR criteria, several were medium- and long-chain FA, such as decanoic, lauric, stearic acid, palmitic, margaric, eladic, octanoic acid, oleic, caproic acid, palmitoleic FA, as well as amino acids, such as beta-alanine and histamine, and the dipeptide carnosine. Further evaluation of the FA profiles of meat may be necessary to understand the relationship between treatments and fatty acid metabolism in cattle.

**Table 5.** Matched metabolites and their mean relative intensity in meat produced from cattle grazing either perennial ryegrass and clover (PRG), a complex multispecies mixture (CMS), or adjacent monoculture strips (AMS).

Metabolites	Method	PRG	CMS	AMS	SD	*P-*value	FDR
Linoleic acid	GCMS	583^a^	680 ^a^	1377^b^	497.6	0.00	0.00
Hypoxanthine	GCMS	13922^a^	16793^a^	23796^b^	7961	0.002	0.14
Pantothenic acid	GCMS	35.6^a^	46.8^a^	76.7^b^	35.51	0.003	0.14
Ribulose-5-phosphate	GCMS	32.8^a^	47.1^a^	69.2^b^	29.79	0.004	0.14
Eicosapentaenoic acid	GCMS	521.6^a^	528.2 ^a^	773.3^b^	249.61	0.09	0.48
Benzoate	Semi-polar Neg	130^b^	83^a^	164^b^	60.5	0.01	0.09
Histidine	Semi-polar Neg	1419^b^	1296^ab^	1218^a^	203.3	0.01	0.09
Carnosine	Semi-polar Neg	43012^b^	39079^ab^	38502 ^a^	4782.9	0.02	0.09
3-Hydroxy benzaldehyde	Semi-polar Neg	130^ab^	95^a^	164^b^	57.9	0.02	0.09
Beta-alanine	Semi-polar Pos	1944^b^	1700^a^	1717^a^	647.9	0.00	0.02
Taurine	Semi-polar Pos	557^a^	593^a^	1273^b^	538.0	0.00	0.05
Palmitocarnitine	Semi-polar Pos	2545^a^	3211^b^	4451^a^	1435.1	0.00	0.06
Hypoxanthine	Semi-polar Pos	11387^a^	13019^a^	14826^b^	2551.3	0.00	0.06
Methionine	Semi-polar Pos	614^b^	470^a^	440^a^	193.3	0.00	0.05
Tyrosine	Semi-polar Pos	421^b^	334^a^	318^a^	103.8	0.01	0.08
Lauroylcarnitine	Semi-polar Pos	995^a^	1223^a^	1985^b^	732.1	0.01	0.09

Metabolites were found to differ (P-value) by treatment the false discovery rate (FDR) is also reported, thresholds < 0.1 indicate ‘statistical significance’, while FDR > 0.1, < 0.25 are ‘possible’).

^a–c^Superscripts within rows that differ are significantly different, *P* < 0.05.

**Figure 3.**
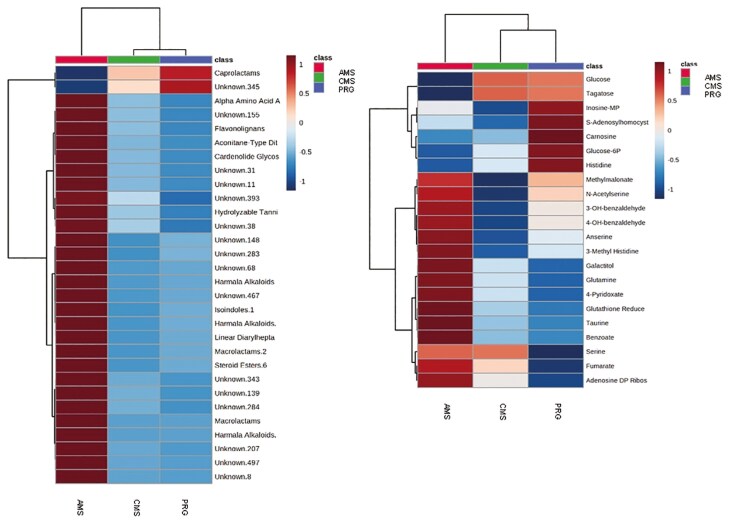
Left: A heat map of the top 30 metabolites identified from untargeted semi-polar metabolomics (positive ionisation), class indicates the tentative compound class, some of which were unidentifiable (null). Right: A heatmap of the top semi-polar metabolites identified from positive ionisation of beef samples collected from cattle grazing either a perennial ryegrass-based diet (MON), a complex multispecies mixture (CMS), or adjacent monocultures (AMS)

A total of 1,053 features were detected in the positive mode of semi-polar metabolomics—this includes unknowns and tentative IDs (untargeted discovery analysis). Two hundred and sixty-seven metabolite features differed between diets. While we were unable to confidently identify many of these features, many were in a retention time range that corresponds to smaller lipids and larger polyphenols (tannins, flavonoids, hydrolysable tannins) as well as alkaloids. Furthermore, these metabolites appear to be greater in AMS beef compared with CMS or PRG. Multivariate analysis of identified semi-polar metabolites yielded from positive ionization found 18 that were significantly different between treatments (raw *P* < 0.05), 8 of which met the FDR threshold with “high confidence” (FDR < 0.1; Table 5). The heat map in Figure 3 presents the treatment differences of these metabolites. Based on Euclidean differences, semi-polar metabolomic profiles of AMS and PRG beef were different, while CMS beef shared similarities with both PRG and AMS. Most of the metabolites were amino acids such as histidine, taurine, leucine, tyrosine, hypoxanthine, and methionine.

One hundred and nineteen metabolite features were identified when semi-polar metabolomics was run in negative mode, including unknown and tentative IDs. Four features differed between diets (Table 5; *P* < 0.1; FDR < 0.1). Metabolites such as histidine and carnosine were also identified by semi-polar metabolomics run in negative ionisation. Relative intensities of benzoate and 3-hydroxybenzaldehyde, which are metabolites related to phytochemicals, increased with AMS compared to PRG. Relative intensities of carnosine and histidine declined with AMS and CMS compared with PRG. These metabolites are associated with beta-alanine metabolism.

**Figure 4.**
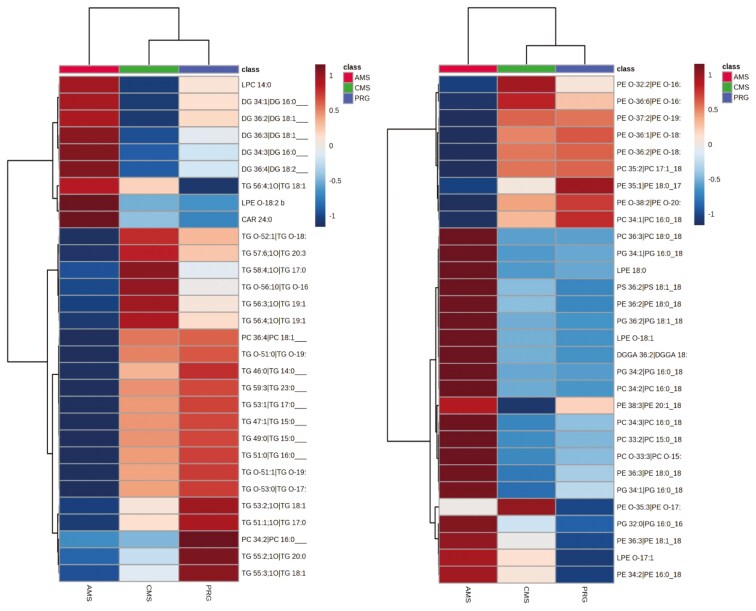
Top 30 lipid features captured from beef in positive (left) and negative (right) ionisation. Beef was produced from three herbage mixtures consisting of perennial ryegrass (PRG), complex multispecies mixture (CMS), or adjacent monocultures (AMS). Lipids are presented as their class abbreviations, and the color legend shows the fold change of lipids in response to diet: Phosphatidylcholine: PC. Lysophosphatidylcholine LPC, Carnitine: CAR, Triacylglyceride: TG, diacylglyceride: DG, Ether-linked phosphatidylethanolamine: EtherPE, Phosphatidylcholine: PC, Lysophosphatidylethanolamine: LPE, Phosphatidylserine: PS, Phosphatidylglycerol: PG, Phosphatidylethanolamine: PE, Cermaide: CER. Sulfatide: HexCer, diacylglycerol: DG, Sulfonolipid: SL, DGGA: diacylglycerol glucuronide.

In total, 3,223 lipid features were detected in positive mode, including unknowns and tentative IDs (untargeted discovery analysis). Based on FDR < 0.1, 258 lipid features differed; however, only 223 were able to be positively matched to the in-house library (Supplementary Table 2) and from these only two features had raw *P*-values *<*0.1, and FDR was “possible” <0.3 and >0. 1. Lipids from a wide range of lipid classes differed between beef coming from different swards treatment, though most appear to be phospholipids and variations on phosphatidylcholine, phosphatidylethanolamine, phosphatidylserine, and phosphatidylglycerol. Diacylglycerols were generally elevated in AMS beef relative to CMS or PRG, while triacylglycerol lipids were often reduced compared with CMS or PRG (Figure 4). While most lipids that were affected by treatment were not identified, hierarchical clustering suggests that lipidomics of CMS and PRG beef showed more similarities than the lipidomic profile produced from the AMS beef.

A total of 887 features were detected in negative mode, 304 lipids differed by diet (*P *< 0.1, FDR < 0.5), and 60 features were highly significant (*P* < 0.05, FDR < 0.1; Figure 4). From negative lipidomic output, 79 features were able to be positively matched to the in-house library, and of these, 24 features differed by treatment (*P *< 0.1, FDR < 0.1); a further 9 features differed by treatment, but FDR thresholds were “hypothetical” (FDR > 0.1 and < 0.2; *P *< 0.1). The AMS treatment reduced ether-linked phosphatidylethanolamines and increased several phosphatidylcholines (PC), phosphatidylglycerol (PG), and lypophosphatidylcholine (LPC) features in meat, compared with either CMS or PRG meat.

## Human Metabolomics

Using GC-MS/MS, 81 metabolites were identified. Correcting for variation of individuals (e.g., sex, age, BMI), and the time postprandial, gamma-tocopherol increased in plasma following consumption of the AMS beef compared with CMS or PRG. In addition, 50 metabolites varied across the time of sampling, but there were no time × treatment interactions (*P *> 0.1). When sex and BMI were included as covariates in the statistical analyses, a total of seven metabolites differed by treatment with a raw *P-*value <0.1. Across the 3- and 5-h samples, gamma-tocopherol (raw *P*-value = 0.001, FDR = 0.09), 3-amino propionic acid (raw *P*-value = 0.01, FDR = 0.46), increased in plasma following consumption of the AMS meat compared with PRG or CMS. Docosahexaenoic acid (*raw P*-value = 0.03, FDR = 0.81) and kynurenine (raw *P-*value = 0.08, FDR = 0.88) increased following consumption of the PRG meat, but FDR exceeded thresholds for hypothetical, and these are reported with very low confidence (FDR > 0.5).

**Figure 5.**
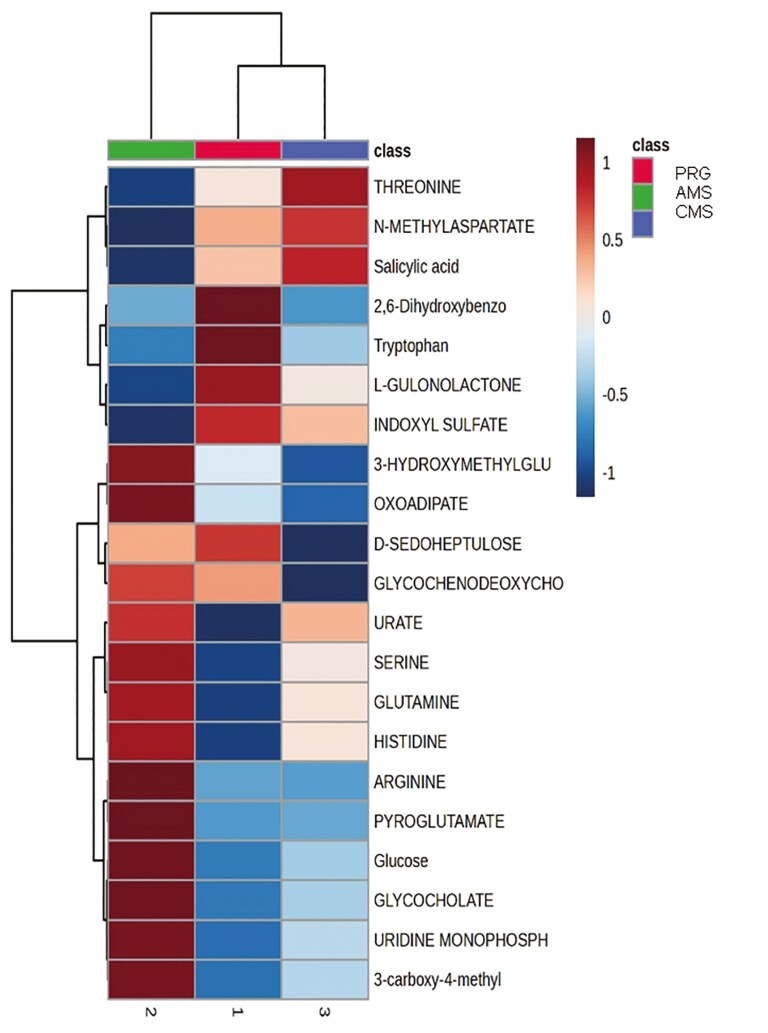
Heatmap of semi-polar metabolomics (negative ionisation) of human plasma following the consumption of beef produced from either a perennial ryegrass pasture (PRG), a complex multispecies mixture (CMS), or adjacent monoculture strips (AMS). Raw *P*-values were <0.1 for 3-hydroxymethylglutarate, indoxyl sulfate, arginine, oxoadipate, and D-sedoheptulose.

Untargeted semi-polar metabolomics yielded 21 metabolites, 7 of which (carnitine, acetoacetate, and phosalone features) were significant (raw *P-*values <0.1), and of those, only one unknown compound had “high confidence” of statistical significance (FDR < 0.1). Semi-polar metabolomics run in positive mode successfully matched 41 metabolites, of which only glycochenodeoxycholate declined (raw *P*-value = 0.05) in participants fed the AMS beef compared with those fed PRG or CMS, although differences exceeded the FDR threshold (FDR > 0.4). Unmatched semi-polar metabolomics yielded 23 features, 10 of which differed by treatment (raw *P*-value < 0.1), and one unknown metabolite met FDR criteria <0.1. From matched semi-polar metabolomics, five features were different across treatments (raw *P*-values <0.1). These were 3-hydroxymethylglutarate (HMG), indoxyl sulphate, arginine, oxoadipate, and D-sedoheptulose (Figure 5, FDR = 0.38). Compared with people fed PRG and CMS beef, AMS participants had a greater relative abundance of HMG, arginine, and oxoadipate, while indoxyl sulphate and D-sedoheptulose declined compared with participants who consumed PRG or CMS meat (Figure 5).

**Figure 6.**
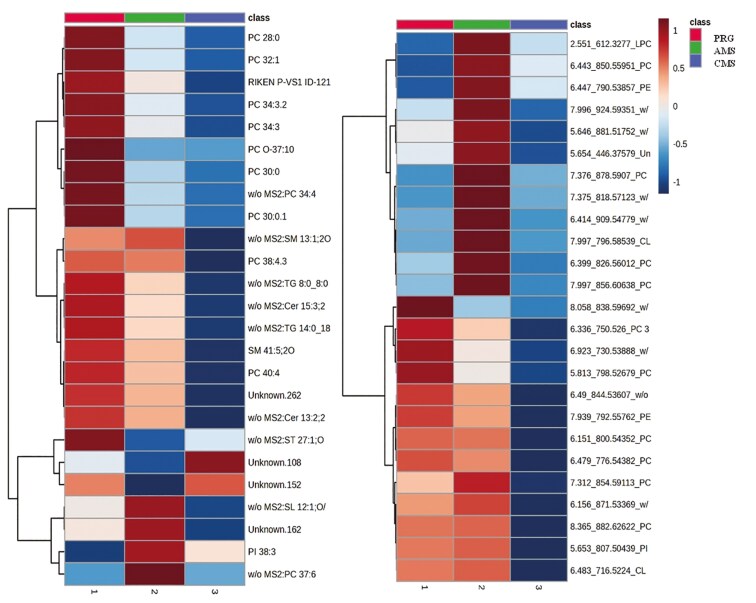
Left: A heatmap of positive lipidomic results. Right: negative profiles of plasma collected from people fed beef produced from either perennial ryegrass and white clover (PRG), a complex multispecies mixture (CMS), or adjacent monoculture strips (AMS).

Lipidomics analyses yielded 1,200 metabolite features in positive ionisation, of which 21 differed with treatment (raw *P *< 0.1, FDR < 0.1). In general, phosphatidylcholine lipids declined following the consumption of AMS and CMS beef compared with PRG (Figure 6). When run in negative mode, 371 features were found, of which 61 were affected by treatment (raw *P *< 0.1). Only six lipids met the FDR criteria (FDR = 0.07). They were variations of phosphatidylcholines and cardiolipins that seemed to be enhanced in the plasma of those fed the AMS beef (Figure 6).

## Discussion

We hypothesized that the taxonomical and phytochemical diversity of plant species would alter the metabolomic and lipidomic profiles of beef and the postprandial metabolomic profiles of human consumers. Our results suggest that metabolomic profiles of beef and human consumers are indeed altered by the type of sward that cattle graze. While changes in the metabolomic profile of human plasma were more subtle due to the limited detection of metabolites, approximately 30% of the metabolites detected were affected in their relative abundances, and several unknown compounds differed by treatment sward.

Metabolomic profiles of the three treatments swards differed, and this affected cattle metabolism. Enrichment ratios suggest that pathways associated with linoleic fatty acid metabolism and eicosapentaenoic acid were upregulated in cattle grazing AMS compared with PRG or CMS, indicating increased presence of long-chain polyunsaturated fatty acids in the AMS swards. Eicosapentaenoic acid is an omega-3 fatty acid usually found in fatty fish such as salmon, but also pasture-raised land animals, and has several health benefits, including antidepressant activities and prevention of coronary heart disease thrombosis—blood clots and arterial plaque formation (Russell and Bürgin-Maunder, 2012). Eicosapentaenoic acid is a long-chain FA that is synthesized from alpha-linolenic acid (ALA), the main omega-3 FA in plants, by the enzyme delta-15-desaturase. ALA is also an essential omega-3 FA with pleiotropic actions that have anti-inflammatory, antidepressant, neuroprotective, and cardioprotective outcomes (Blondeau et al., 2015). While ALA is reported to reduce circulating LDL-C, Blondeau et al. (2015) point out that positive responses have been reported from chronic ALA supplementation. Subtle changes to plasma, EPA, and omega-3 FA have been reported after 4 wk of consumption of either grain- or pasture-finished beef or 6 mo of consumption of eggs and chicken meat enriched with algae-sourced omega-3-PUFA (McAfee et al., 2011; Stanton et al., 2020). Therefore, the short-term nature and potentially low dosage of the current study may not have been sufficient to generate a response in omega-3 FA in human consumers.

The AMS treatment also increased the relative abundance of PA (vitamin B5) in beef. Vitamin B5 is an essential nutrient involved in the synthesis of coenzyme A—needed for FA synthesis and degradation. Supplementation of PA reduced cholesterol levels by 17% after 4 wk in people suffering from dyslipidaemia (high LDL, TGL, and total cholesterol) (Chen et al., 2015). The long-term supplementation of PA is inversely correlated to CRP levels and has been suggested to reduce low-grade inflammation associated with early stages of heart disease (Chen et al., 2015). In addition, 3-hydroxybenzaldehyde (3HBA) also increased in beef produced with the AMS. Benzaldehydes are commonly found in nature, and 3HBA has an OH group at the meta-position of the phenol ring, and it is a potent intracellular antioxidant (Bortolomeazzi et al., 2007; Chen et al., 2021). Research in mice has identified that 3HBA is vasoprotective, preventing angiogenesis and adenosine diphosphate—a platelet agonist of thrombosis formation (Kong et al., 2016). Evidence suggests 3HBA is antimutagenic (Gustafson et al., 2000), cytotoxic (Tseng et al., 2001), has antibacterial functions (Chen et al., 2021), and can even influence flavour (Bortolomeazzi et al., 2007). Vitamin B5 and 3HBA both have vasoprotective, anti-inflammatory, and “heart-healthy” roles that were upregulated in the AMS beef, which could have human health benefits compared with traditional PRG beef, highlighting the need for longer-term clinical research.

The results suggest that herbage composition grazed by cattle alters the human metabolomic profile following a single meal of beef. Gamma-tocopherol (γ-tocopherol) was elevated in the AMS sward, and this increased the circulating concentrations of γ-tocopherol in people following the consumption of AMS beef. Others have identified elevated tocopherol levels in bison finished on an extensively grazed pasture-based diet, compared to those fed a total-mixed ration diet (van Vliet et al., 2023). Quantitative comparisons between the current dataset and this work are not possible, but highlight a direction for future research. Gamma-tocopherol is the second most common form of vitamin E, a lipophilic molecule (second to α-tocopherol) that is one of eight isomers. It is the most effective anti-inflammatory, due to its ability to inhibit cyclooxygenase and 5-lipoxygenase activity, and antioxidant through capture of lipophilic electrophiles (Jiang et al., 2001). The content of γ-tocopherol was observed to be numerically greater in the relative abundance of the chicory plant compared with all other plants or plant mixes. Elevated γ-tocopherol content has been reported in *Chicorium spinosum* (spiny chicory), which belongs to the same family Ateraceae, as the *C. intybus* used in the current study. Research on γ-tocopherol has also indicated that it is superior to α-tocopherol in its ability to reduce cancer cell growth, trapping reactive N-species through the formation of 5-nitro-γ-tocopherol (Jiang et al., 2022). A quantitative description of the vitamin content of pastures, combined with grazing practices, is needed to understand the potential role or benefit to human consumers. However, the clear transfer from pasture to human supports our initial hypothesis.

The human results also presented five different (*P*-values *<*0.1) features between treatments. These were 3-hydroxymethylglutarate (HMG), indoxyl sulphate, arginine, oxoadipate, and D-sedoheptulose, with their FDR locating them as “possible.” Compared with people eating PRG and CMS beef, AMS participants experienced a greater relative abundance of HMG, arginine, and oxoadipate, while indoxyl sulphate and D-sedoheptulose declined (Figure 5). Indoxyl sulphate is produced from the breakdown of tryptophan by colon microbes—and may reflect the exogenous intake of phenols and indoles (Evenepoel et al., 2009; Schepers et al., 2010). In patients with chronic kidney disease, it is considered a uremic toxin, but it is also reported to vary considerably between individuals, increasing with the consumption of meat compared to a vegetarian diet (Patel et al., 2012). Accumulation of indoxyl sulphate is also associated with additional side effects in renal, skeletal, and cardiovascular systems (Evenepoel et al., 2009; Schepers et al., 2010). Arginine is an amino acid required to help build muscle and rebuild tissue. The body also converts this amino acid into nitric oxide, which is a vasodilator (Preli et al., 2002). These results not only need further investigation but also highlight the need for longer-term human health studies.

Metabolomic profiling offers insight into changes in biochemical pathways and small metabolites fundamental to basic metabolism. While changes in the relative abundance of metabolites indicate that some pathways may be upregulated or downregulated in response to the inclusion of functionally diverse AMS swards, further analysis in other laboratories with broad metabolomic and lipidomic libraries and known standards is needed to identify, quantify, and compare the current results. The untargeted LC-MS/MS analyses revealed 267 metabolites in human plasma differed by treatment, while we were unable to identify these lipids, they still support our initial hypothesis that sward composition influences consumer metabolomic profile. There is a need to continue developing diverse pasture systems based on metabolomic profiling of not only the livestock grazing diverse feeds but also the human consumer.

Several interactions between treatment and date were identified across plant and animal measurements. While cattle were allocated similar amounts of herbage and estimated DMI was similar across treatment, DMI of the CMS cattle was numerically less than PRG or AMS, and this most likely explains the lower LW and LWG achieved from this treatment. Furthermore, CMS herbage contained lower quantities of CP, and greater content of NDF, ADF, and DMD tended to be less than AMS or PRG, which likely explains the reduced performance of CMS cattle. Plasma concentrations of NEFA declined 29% in cattle grazing CMS compared with AMS or PRG herbages. The plasma concentrations of NEFA reflect fatty acids produced in the liver from adipose reserves within the animal. Therefore, the lower concentration of NEFA in the CMS treatment may reflect lower caloric intake and rumen digesta outflow, reducing nutrient supply to the host animals and thereby average LWG.

Treatment-by-time interactions for livestock performance (LW and LWG) were observed across all treatments, reflecting seasonal variation of plant growth and chemical components such as CP and ME content. The AMS diet contained less CP and was generally lower in WSC and DMD than PRG, reducing the nutritional value of the diet during late summer before animal slaughter (February). However, the chemical composition and growth of the AMS sward were favorable for liveweight growth and performance during late Spring and early Summer (November to January), which maintained the average LWG and LW of the cattle grazing this sward when averaged across the experimental period. Seasonal variation of herbage growth and chemical composition (including phytochemical components) of forages and forbs is needed to understand performance outcomes and farm-system profitability. Seasonal fluctuations in plant chemical composition and phenological state will also impact metabolomic profiles and subsequent impacts on livestock and human consumers. Many plant phytochemicals are synthesised in response to abiotic and biotic stress and during the reproductive growth phase (Fleming and Gregorini, 2023). The current study evaluated metabolomic profiles from herbage and cattle during summer months when many plant species are in a reproductive phenological state. However, plant synthesis of phytochemicals does occur during vegetative stages of growth, which again emphasizes the need for grazing research on a greater scale and longer duration.

Carcass characteristics and beef quality were assessed using the Silver Fern Farms Eating Quality System. Meat color increased in cattle that grazed the AMS swards, compared with those grazing PRG or CMS. The meat color of the chilled rib eye muscle area was scored against a set of color reference standards, with scores increasing relative to the darkness of the meat color. Meat color reflects myoglobin levels and ultimate pH, as postmortem glycolysis decreases muscle pH, resulting in a brighter color of meat that is preferred by consumers. If the ultimate meat pH is high, the meat proteins will associate with more water in the muscle, and therefore fibres will be tightly packed, resulting in meat that is “tough.” However, the ultimate pH in AMS cattle was similar to PRG or CMS cattle. The numerical difference between herbage treatments was minor (0.2 to 0.3) and did not alter the overall grade of the carcass. Thus, carcass differences are unlikely to be financially meaningful but require further study.

The results of this study indicate that livestock performance can be maintained using AMS when used strategically throughout the finishing period compared to traditional herbages such as PRG. Further development of the plant species and their level of inclusion in the sward, along with consideration of their seasonality relative to livestock metabolic requirements, is needed to further develop a more consistent response in both performance and metabolomic profiles. However, this research suggests that a more functional approach to integrating plant diversity in pastoral grazing systems through adjacent swards may have additional benefits to the biochemical composition of the meat, which is passed on to the consumer. The AMS diet contained areas of concentrated plant species that enabled cattle to graze each forage selectively, and this likely explains the accumulation of certain metabolites found in beef and the consumer. In comparison, the CMS herbage contained a greater number of pasture plants, but these were all mixed, which, at each bite, would hinder cattle from making discrete decisions on what they are grazing. While grazing behavior was not formally analyzed, we hypothesise that the CMS and even PRG herbages would prevent cattle from formulating their own diets based on internal and external cues and post-ingestive feedback mechanisms, as has been observed elsewhere (Provenza, 1995; Provenza, 1996; Forbes and Provenza, 2000; Villalba and Provenza, 2007; Catanese et al., 2013; Konagh Garrett et al., 2021). Livestock performance on AMS was greater during early to mid-summer months, suggesting that adjacent forages could be developed to enhance livestock performance. The limited number of animals used in this study is also likely a factor impacting the production response, and studies with a greater number of animals and replication across mobs (as opposed to the individual animal used currently) will provide a better indication of the impact on livestock performance.

## Conclusions

Metabolomic profiles of the human consumer reflect not only beef finished from different forages but also provide evidence that consumer metabolism and potential health outcomes reflect herbage metabolome and grazing management. Based on a range of targeted and nontargeted approaches, the metabolomes of cattle that grazed a functionally diverse sward—AMS—differed from those that grazed either a PRG or a CMS mixture containing a greater relative abundance of metabolites, which may benefit human health. Furthermore, longer-term clinical studies are needed to evaluate potential human health advantages further. This research is the first to identify metabolomic linkages between herbages, the beef produced from grazing these herbages, and the human consumer.

## Supplementary Material

skaf254_suppl_Supplementary_Materials_1

## Data Availability

Supporting data relating to human variables is available on request, but has not been shared to an open repository to maintain the privacy of the participants involved in this research. Variables of herbage and beef are available on GitHub (https://github.com/AnitaFleming/Connecting-metabolomes).

## Abbreviations

3HBA: 3-hydroxybenzaldehyde
ALA: alpha-linolenic acid
AMS: adjacent monoculture strips of forages
CMS: swards of complex multispecies of grasses, legumes, and forbs
FA: fatty acids
FDR: false discovery rate
HMG: hydroxymethylglutarate
LWG: liveweight gain
NEFA: non-esterified fatty acids
PA: pantothenic acid
PRG: swards of perennial ryegrass × white clover
PSM: plant secondary metabolites
PUFA: poly unsaturated fatty acid
PUN: plasma urea nitrogen
TAS: total antioxidant status

## Code availability

Statistical coding for analysis of beef finishing data is available at https://github.com/AnitaFleming/Connecting-metabolomes.
